# Bilateral but not unilateral tubal obstruction is associated with positive chlamydia serology

**DOI:** 10.5935/1518-0557.20190049

**Published:** 2020

**Authors:** Fabiana C Approbato, Mário S Approbato, Mônica C S Maia, Yanna A R de Lima, Maria A Barbosa, Beatriz B do C Benetti

**Affiliations:** 1 Human Reproduction Laboratory. Obstetric and Gynecology Dept. Federal University of Goias State, Brazil; 2 Nursing School. Federal University of Goias State, Brazil; 3 Nutrition School. Federal University of Goias State, Brazil

**Keywords:** tubal obstruction, Chlamydia trachomatis, C. trachomatis, tubal patency, infertility

## Abstract

**Objective:**

To assess the association between positive *Chlamydia trachomatis (C. trachomatis)* serology and unilateral or bilateral tubal obstruction.

**Methods:**

This was a cross sectional study that evaluated the association of positive *C. trachomatis* serology (Immunofluorescence Indirect Serology, IIF or Enzyme Immune Essay, EIE), in two infertile groups: A. 243 patients (27 with unilateral obstruction and 216 without it). B. 247 patients (31 with bilateral obstruction and 216 without it). The exclusion criteria were tubal ligation (tubectomy) and tubal surgery. The statistical test (SPSS 17.0) was the Chi-Square with a *p*=5%. Tubal obstruction was diagnosed through hysterosalpingography (HSG).

**Results:**

The mean age of the patients without obstruction was 33.6 years, SD 4.9. The mean age of the patients with unilateral obstruction was 33.7 years SD 4.9. The mean age of the patients with bilateral obstruction was 33.6 years, SD 4.9. There was no statistically significant difference between the age groups. In group A (unilateral obstruction versus serology) the Chi-Square was 0.02 (*p*=n.s.) and the Attributable Risk (AR) = 0.7%. In Group B (bilateral obstruction versus serology) the Chi-Square test was 9.87 (*p*<0.005) and the AR = 14.8%.

**Conclusion:**

This study found a strong and statistically significant association between bilateral tubal obstruction and *C. trachomatis* positive serology. The power of the test was 86%. There was no association between unilateral obstruction and positive serology.

## INTRODUCTION

*Chlamydia trachomatis* infection is one of the most common sexually transmitted bacterial diseases, particularly in women (WHO, 2001). In 2009, a total of 1,244,180 *Chlamydia* infections was reported to the Centers for Disease Control (CDC) in 50 states and the District of Columbia, an increase of 2.8% when compared with 2008 (CDC, 2010). Even in developed countries the prevalence of *C. trachomatis* seems to be increasing. In Sweden, the incidence of *Chlamydia* infection increased from 171.7 to 406.2 cases per 100,000 between 1998 and 2009 (Sylvan & Christenson, 2008; Christenson & Sylvan, 2011).

The risk ratio of infection-induced reproductive diseases can be underestimated because up to 70%-80% of acute *Chlamydia* infections in women are asymptomatic or subclinical, and are not diagnosed or treated (Peipert, 2003). According to the World Health Organization, 10%-40% women with untreated or repeated infections develop symptomatic pelvic inflammatory disease (WHO, 2016), which results in scarring and fibrosis of the Fallopian tubes (Hillis *et al*., 1997) and can lead to ectopic pregnancy (Chow *et al.,* 1990). Moreover, 30%-40% of cases of female infertility are caused by postinfectious tubal damage resulting in hydrosalpinx (Brunham & Rey-Ladino, 2005; WHO, 2016). *Chlamydia* infection treatment does not always prevent progressive tubal damage (Brunham & Rey-Ladino, 2005).

*C. trachomatis* infection has been on the rise worldwide and frequently causes tubal damage, often irreparable, of difficult management, limiting the reproductive capacity of women (WHO, 2001). Due to its serious consequences, *C. trachomatis* antibody testing is part of the infertility work-up suggested by the Dutch Society of Obstetrics and Gynecology (Dobekousen *et al*., 1994).

The prevalence of tubal pathology in female infertility varies depending on the author. Some published that it is estimated that 30% of infertile females have tubal pathology (Evers, 2002; Brandes *et al*., 2010). Dobekousen *et al*. (1994) found a total of 14% of tubal factor in female infertility. Between 10 and 15% of all women seeking *In Vitro* Fertilization in the United Kingdom are due to tubal infertility (HFEA, 2016).

Several hypotheses about tubal implantation have been proposed, including inhibition of ciliary beating and muscle contraction, stimulation of tubal secretion, and early embryo-tubal cell interaction (Shah *et al.*, 2005). It has been speculated that an antibody response to the *Chlamydia* 60-kDa heat shocks the protein (hsp-60) and causes a tubal inflammatory response, leading to tubal blockage or a predisposition to tubal implantation (van Mourik *et al*., 2009; Daponte *et al*., 2012). There is evidence that the steroid hormones 17β-estradiol (E_2_) and progesterone (P_4_) increase susceptibility to *Chlamydia* infection and modulate inflammation in epithelial cells (Amirshahi *et al*., 2011; Hall *et al*., 2011).

The tubal secretory function is incompletely characterized, but epithelial cell secretions are known to affect gamete fertilization and early human embryo development. The makeup and volume of Fallopian tube fluid depend on physiological and pathophysiological conditions (Avilés *et al*., 2010). One consequence of tubal infection in mice is hydrosalpinx, as defined by tubal dilatation and abnormal fluid buildup (Shah *et al*., 2005). Hydrosalpinx has adverse effects on ongoing pregnancies and female fertility, perhaps by reducing endometrial receptivity (Cakmak & Taylor, 2011). Since successful intrauterine implantation requires a sustainable microenvironment (Jabbour *et al*., 2006), an important question is how *Chlamydia*-induced hydrosalpinx formation changes the local microenvironment and consequently triggers tubal implantation in women.

We did not find publications investigating whether or not unilateral tubal obstructions are associated with seropositivity for *C. trachomatis* seropositivity. It was the goal of this paper to evaluate if seropositivity also relates to unilateral tubal obstruction.

## MATERIALS AND METHODS

This was a cross-sectional study that evaluated the association of positive *Chlamydia* serology (Immunofluorescence Indirect Serology, IFI or Enzyme Immune Essay, EIE), in two infertile groups: Group A: 243 patients (27 with unilateral and 216 without tubal obstruction). Group B: 247 patients (31 with bilateral and 216 without tubal obstruction). The exclusion criteria were tubal ligation (tubectomy) and tubal surgery. The statistics assessment package used was the SPSS 17.0. The Chi-Square test yielded *p*=5%. Tubal obstruction diagnosed was made by hysterosalpingography, which is a test part of the work-up of infertile couples, a minimally invasive method of evaluating tubal patency and is performed as the first line approach for assessing tubal pathology (Foroozanfard & Sadat, 2013).

## RESULTS

**Figure 1:**
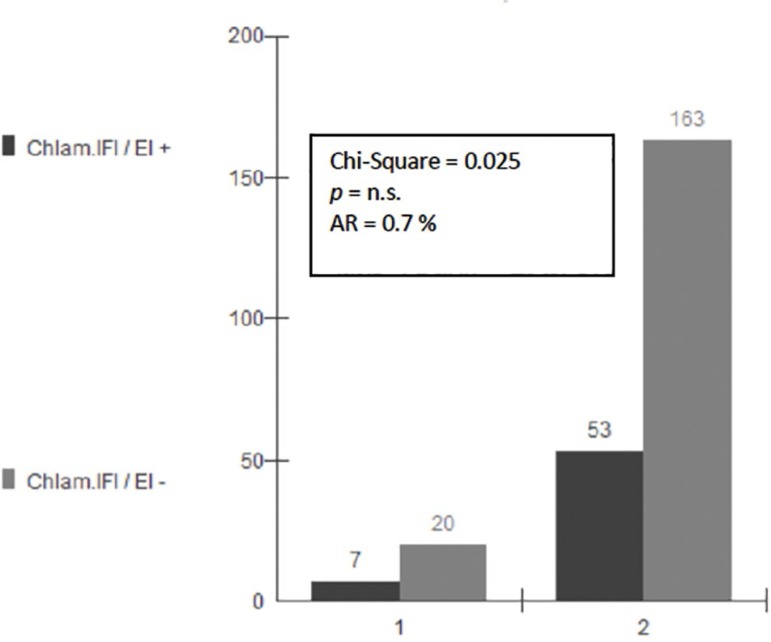
Unilateral Tubal Obstruction and *Chlamydia* Seropositivity. Human IVF Laboratory. Federal University of Goiás State, Brazil, 2018. 1 = Obstruction Yes. 2 = Obstruction No. AR = Attributable Risk. IFI = Indirect Immunofluorescence. EI = Enzyme immunoassay

**Figure 2:**
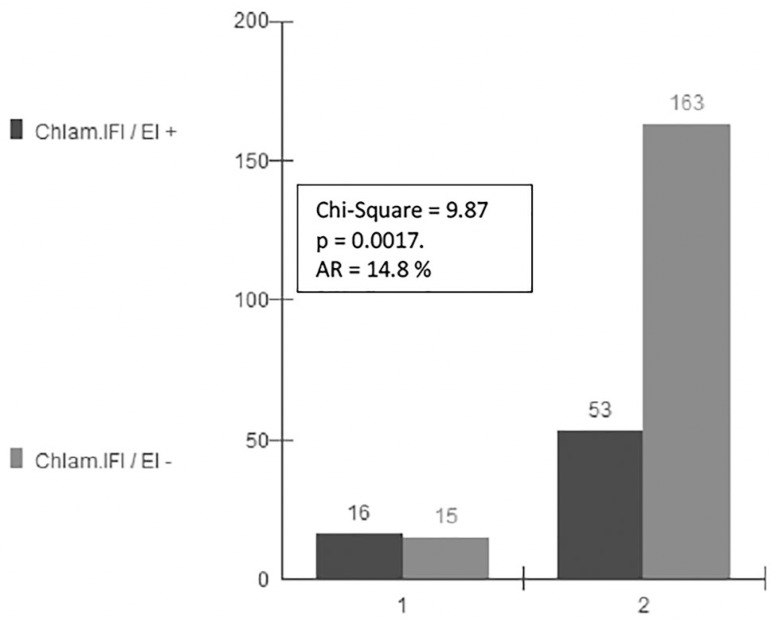
Bilateral Tubal Obstruction and *Chlamydia* Seropositivity. Human IVF Laboratory. Federal University of Goiás State, Brazil, 2018. 1 = Obstruction Yes. 2 = Obstruction No. AR = Attributable Risk. IFI = Indirect Immunofluorescence. EI = Enzyme immunoassay

The mean age of the patients without obstruction was 33.6 years, SD 4.9. The mean age of the patients with unilateral obstruction was 33.7 years, SD 4.9. The mean age of the patients with bilateral obstruction was 33.6 years, SD 4.9. There is no statistical difference between age groups. In group A (unilateral obstruction versus serology) the Chi-Square was 0.02 (*p*=n.s.), and the Attributable Risk (AR)=0.7% (Figure 1). In Group B (bilateral obstruction versus serology) the Chi-Square test was 9.87 (*p*<0.005), with AR=14.8% (Figure 2).

## DISCUSSION

HSG is used worldwide to evaluate tubal patency. It is a simple method for assessing female sterility, it is a less expensive and elementary method to evaluate tubal pathologies, and can identify some congenital uterine anomalies. The advantage of laparoscopy is that it can identify some other pelvic abnormalities which may be the cause of infertility that cannot be detected by HSG, such as endometriosis, adhesions and tuberculosis. However, one limitation of HSG is that the interpretation of the images depends on the experience and skill of the radiologists involved (Foroozanfard & Sadat, 2013).

In a research, HSG was compared with laparoscopy, and the results showed that sensitivity was 65% for tubal patency, but it increases the achievement of spontaneous pregnancy by three fold (Torre *et al.*, 2010). Other authors in a cohort study investigated eighty-two infertile cases to compare tuboperitoneal factors by HSG and laparoscopy, and the results showed that pathological findings were seen in 45.1% by HSG and 65.85% by laparoscopy. The sensitivity and specificity of HSG were 63% and 89.3%, respectively, and the positive predictive value was 92%, with a 55% negative predictive value, and an accuracy ratio was 72% (Sakar *et al*., 2008).

In a series of 360 infertile women, an initial hysterosalpingography study suggested an incidence of unilateral proximal tubal obstruction in 18, and bilateral obstruction in 22 women. When the HSG was repeated one month later, the unilateral obstruction persisted in 12 (3.3%) and the bilateral obstruction in 9 (1.1%) women (Dessole *et al*., 2000).

In the year 2018, the prevalence of tubal obstruction in a sample of 292 infertile patients seen at the Humana Laboratory of the Federal University of Goiás State had the following distribution: Uni or bilateral obstruction in 21.9%, bilateral in 10.9% and unilateral in 9.9%. Serology (Immunofluorescence Indirect Serology, IIF or Enzyme Immune Assay, EIA) was positive for *C. trachomatis* in 27.1%. PCR was positive in 0.94% of patients. Steiner *et al*. (2015) published a study using a newer anti-Ct (anti *Chlamydia trachomatis*) assay developed by Geisler *et al.* (2012) that is, an elementary body-based ELISA that has been shown to have higher sensitivity and specificity than prior assays. Steiner *et al.* (2015) found that 19% of the women in their sample were seropositive for anti-*C. trachomatis* IgG3. Approbato (2012) found in a Master of Science (MS) graduate paper that 0.83% (120 patients) were PCR positive for *C. trachomatis*. In this same publication, this author found 36.5% of seropositivity in IIF or EIA.

Even in the presence of tubal patency, anti-*C. trachomatis* IgG3 seropositivity is associated with a lower likelihood of pregnancy and increased pregnancy complications. Steiner *et al.* (2015) found that positive anti-*C. trachomatis* diagnosis, using the new IgG3 test, women have as high as 3 times the risk of having an ectopic pregnancy. These same authors found that Anti-*C. trachomatis* IgG3 seropositive women were significantly less likely to conceive (risk ratio [RR] 0.65, 95% confidence interval [CI] 0.52-0.83) or to have a live birth (RR 0.59, 95% CI 0.43-0.80). To date, the specificity of the anti-Ct assay due to cross-reactivity with other *Chlamydia* strains has limited its clinical utility as a predictive test. Standard methodologies used to assess Fallopian tubes, assess the patency but not function (Feinberg, 2015). The utility of the improved detection method for anti-Ct antibody should be met with cautious, because tubal patency does not imply tubal function. Seropositive patients should not be driven immediately for IVF treatments. But, it may be reasonable, in the setting of positive anti-Ct antibodies, to limit the number of ovulation induction cycles and pursue IVF if not pregnant after three cycles (Feinberg, 2015).

Titration of anti-*C. trachomatis* antibodies has been used to track tubal obstruction and pathologies. Rodgers *et al.* (2011) published that infertile women with laparoscopically identified tubal pathologies developed significantly higher titers of anti-*C. trachomatis* antibodies. Many publications (Musich & Behrman, 1983; Serafini & Batzofin, 1989; Healy *et al*., 1994) described a prevalence of tubal obstruction and others pelvic diseases from 25% to 35% in patients with infertility, mainly by *C. trachomatis*. Nevertheless, there's no evaluation if these tubal obstructions were uni or bilateral. This paper is one of the first to show that unilateral obstruction is not related to seropositivity for *C. trachomatis*. It may be that in these cases, tubal spasms or false positivity are the probable causes. It seems that when there is an infection by this sexually transmitted disease, it affects both female tubes simultaneously. Other studies should be carried out to confirm these finds.

## CONCLUSION

This study found a strong and statistically significant association between bilateral tubal obstruction and *Chlamydia* positive serology. The power of the test was 86%. There was not an association between unilateral obstruction and positive serology. This is one of the first published studies to show this.

## ACKNOWLEDGEMENT

The authors would like to thank the Human Reproduction Laboratory HC/GO to support this study.

This study was partially financed by the Coordenação de Aperfeiçoamento de Pessoal de Nível Superior - Brasil (CAPES) - Finance Code 001.
